# Effects of regorafenib on the mononuclear/phagocyte system and how these contribute to the inhibition of colorectal tumors in mice

**DOI:** 10.1186/s40001-023-01099-2

**Published:** 2023-04-03

**Authors:** Sylvia Grünewald, Maria Stecklum, Manuel Rizzo, Jonathan Rathjens, Lukas Fiebig, Dieter Zopf

**Affiliations:** 1 Bayer AG, Berlin, Germany; 2 Present Address: Nuvisan ICB GmbH, Berlin, Germany; 3 EPO Berlin-Buch GmbH, Berlin, Germany; 4 Chrestos Concept GmbH & Co. KG, Essen, Germany

**Keywords:** Colorectal cancer, Regorafenib, CSF1R, CCL2, Tumor-associated macrophages, Preclinical

## Abstract

**Background:**

Regorafenib was previously shown to reduce tumor-associated macrophages and potently inhibit colony-stimulating factor 1 receptor (CSF1R), also known as CD115, in biochemical assays. The CSF1R signaling pathway is essential in the biology of the mononuclear/phagocyte system, which can promote the development of cancer.

**Methods:**

A deeper investigation of regorafenib’s effects on CSF1R signaling was performed using preclinical in vitro and in vivo studies with syngeneic CT26 and MC38 mouse models of colorectal cancer. Peripheral blood and tumor tissue were analyzed mechanistically by flow cytometry using antibodies against CD115/CSF1R and F4/80 and by ELISA for chemokine (C–C motif) ligand 2 (CCL2) levels. These read-outs were correlated with drug levels for the detection of pharmacokinetic/pharmacodynamic relationships.

**Results:**

Potent inhibition of CSF1R by regorafenib and its metabolites M-2, M-4, and M-5 was confirmed in vitro in RAW264.7 macrophages. The dose-dependent growth inhibition of subcutaneous CT26 tumors by regorafenib was associated with a significant reduction in both the number of CD115^hi^ monocytes in peripheral blood and the number of selective subpopulations of intratumoral F4/80^hi^ tumor-associated macrophages. CCL2 levels were not affected by regorafenib in blood but increased in tumor tissue, which may contribute to drug resistance and prevent complete tumor remission. An inverse relationship between regorafenib concentration and the number of CD115^hi^ monocytes and CCL2 levels was observed in peripheral blood, supporting the mechanistic involvement of regorafenib.

**Conclusions:**

These findings may be clinically useful in optimizing drug dosing using blood-based pharmacodynamic markers and in identifying resistance mechanisms and ways to overcome them by appropriate drug combinations.

**Supplementary Information:**

The online version contains supplementary material available at 10.1186/s40001-023-01099-2.

## Background

Regorafenib (REG) is an orally administered small-molecule multikinase inhibitor widely approved for the treatment of advanced colorectal cancer (CRC), gastrointestinal stromal tumor (GIST), and hepatocellular carcinoma (HCC) [1–3]. REG as a single agent has been shown to improve overall survival in patients with CRC or HCC and progression-free survival in patients with GIST. REG exerts antitumor activity by various mechanisms, including inhibition of angiogenesis, tumor cell proliferation and metastasis, induction of apoptosis, and modulation of tumor immunity. These activities are mediated by blocking multiple protein kinases, including vascular endothelial growth factor (VEGF) receptor (VEGFR) 1–3, stem cell factor receptor (KIT), platelet-derived growth factor receptor α and β, fibroblast growth factor receptor, rearranged during transfection receptor, wild-type and mutant (V600E) rapidly accelerated fibrosarcoma 1 kinase (B-RAF), and colony-stimulating factor 1 (CSF1) receptor (CSF1R), also known as CD115 [4, 5].

To improve the activity of REG, it has been combined with other cancer therapeutics including immune checkpoint inhibitors [6–8]. This was encouraged by the previously reported immunomodulatory effects of REG in preclinical models of CRC, HCC, and melanoma. In syngeneic models of CRC and HCC, REG was shown to reduce tumor-associated macrophages (TAMs), which are considered to enhance tumor growth [9–11], and to promote macrophage polarization toward the antitumorigenic M1 subtype, thereby increasing the M1:M2 ratio [11, 12]. M2 macrophages secrete chemokine (C–C motif) ligand 2 (CCL2)/monocyte chemoattractant protein-1 (MCP-1) [11], which promotes tumor growth by binding and activating C–C chemokine receptors type 2 (CCR2), type 4 (CCR4), and type 5 (CCR5) [13] expressed on certain immune cells, endothelial cells, cancer-associated fibroblasts, and tumor cells. In patients with CRC, increased CCL2 expression was associated with worse outcomes [14] and resistance to VEGF/VEGFR2 inhibition by bevacizumab [15]. In a small cohort of patients with metastatic CRC, circulating levels of CCL2 at baseline were significantly higher in nonresponders compared with healthy controls and tentatively higher compared with responders [16].

In addition, intratumoral regulatory T cells were depleted upon REG treatment in a syngeneic orthotopic CT26 CRC tumor model [12]. Similarly, patients with gastric cancer who responded to REG plus anti-programmed cell death protein-1 therapy showed reduced levels of intratumoral regulatory T cells [6]. In the RIL-175 HCC cell line, REG induced CXCL10 expression, thereby promoting the infiltration of CD8^+^CXCR3^+^ cytotoxic T lymphocytes into RIL-175 tumors. CXCL10 was also elevated in plasma samples of patients with HCC or acute myeloid leukemia after 2 weeks of REG treatment [17]. Correspondingly, REG was found to induce CXCL10 expression in Huh7 and HepG2 cell lines [18]. In HCC models, REG in combination with immune checkpoint inhibitors was more effective at an intermittent dose, indicating that an optimal dose for immunomodulatory effects, which is different from the dose required for optimal single-agent antitumor activity, exists for REG [11, 17]. REG has also been identified as a potent inhibitor of interferon-γ-induced expression of programmed death-ligand 1 and indoleamine 2,3-dioxygenase in a preclinical melanoma model, suggesting another immunomodulatory effect of REG [19].

We have previously shown that REG, and its major human metabolites M-2 and M-5, potently inhibit human recombinant CSF1R kinase in a biochemical competitive binding assay, with dissociation constants between 13 and 27 nM [5]. CSF1R and its ligands macrophage CSF1 and interleukin-34 play an important role in the mononuclear/phagocyte system [20]. Because REG reduced the number of TAMs, it was of interest to further investigate REG’s role in CSF1R signaling and the consequences on monocytes and macrophages in peripheral blood (PB) and CRC tumors. As selective CSF1R inhibitors, such as pexidartinib, were shown to reduce the number of CD14^dim^CD16^+^ blood monocytes in patients with glioblastoma [21], this paper explores whether REG, which also inhibits CSF1R, has a similar effect.

## Methods

### Materials

REG, M-2, M-4, and M-5 were provided by Bayer AG. RAW264.7 (TIB-71) and CT26.WT (CRL-2638) cells were from the American Type Culture Collection. MC38 cells were from the National Cancer Institute. The syngeneic CT26 (classified as microsatellite stable) [22] and MC38 (classified as microsatellite instable) [23] CRC mouse cell lines were derived from carcinogen-induced BALB/c and C57BL/6 mice, respectively [24, 25].

For cell culture experiments, drugs were dissolved and diluted in 100% dimethylsulfoxide (DMSO) prior to a final dilution of 1:200 in the culture medium, resulting in working drug concentrations and 0.5% DMSO. For in vivo application, REG was dissolved in polypropylene glycol, polyethylene glycol 400 (PEG400), Pluronic F68, and water (34:34:12:20) (vehicle V1) or in PEG400 and 125 mM methanesulfonic acid in water (80:20; v/v) (vehicle V2). Details of antibodies and polymerase chain reaction (PCR) probes are provided in the Methods in Additional file 1.

### CSF1R inhibition in RAW264.7 cells

RAW264.7 cells (10^6^) were seeded into cell culture dishes 6 cm in diameter and incubated in 4 mL RPMI/10% fetal bovine serum (Biochrom) for 24 h at 37 °C in a humidified 5% CO_2_ atmosphere. Cells were washed once with phosphate-buffered saline (PBS) and starved in 3 mL RPMI/0.1% bovine serum albumin for another 24 h. Cells were then treated for 1 h at 37 °C with drugs at the indicated final concentrations, with 0.5% DMSO as vehicle control, or left untreated, and finally stimulated for 2 min at 37 °C with 100 ng/mL recombinant mouse CSF1 (R&D Systems) by adding 1 mL prewarmed starving medium containing 400 ng/mL CSF1. Dishes were placed on ice and cells were immediately washed with ice-cold PBS and lysed with 50 µL lysis buffer (50 mM HEPES pH 7.5, 150 mM NaCl, 1.5 mM MgCl_2_, 10% glycerol, 1% Triton X-100, 0.5% desoxycholate, 0.2% sodium dodecyl sulfate, 1 mM ethylene glycol-bis[β-aminoethyl ether]-N,N,N′,N′-tetraacetic acid, 100 mM NaF, 10 mM Na_4_P_2_O_7_, 1 mM Na_3_VO_4_, 1% Phosphatase Inhibitor Cocktail 3 [Sigma-Aldrich], 1 mM phenylmethyl sulfonyl fluoride, Complete Protease Inhibitor Cocktail [Roche CustomBiotech; 1 tablet/10 mL], and 50 U/mL Benzonase [Sigma-Aldrich]). Lysed cells were scraped into Eppendorf tubes, incubated on ice for 20 min, and centrifuged at 4 °C for 10 min at 13,000 rpm; supernatants (lysates) were transferred into new tubes and stored at −80 °C.

### Western blotting

Protein concentrations were determined using Pierce bicinchoninic acid assay (Thermo Fisher Scientific) and 40 µg/lane of total lysate was separated by SDS-PAGE on 4–12% Criterion XT Bis–Tris Gels (Bio-Rad) and subsequently transferred to polyvinylidene difluoride membranes using the Trans Blot Turbo Blotting System (Bio-Rad). For antigen detection, membranes were blocked with PBS Odyssey blocking reagent (LI-COR Biosciences) for 1 h at room temperature (RT). Primary antibodies were incubated overnight at 4 °C and the fluorescent secondary antibodies were incubated for 1 h at RT. After each antibody incubation, blots were washed with PBS with 0.05% Tween 20 (Carl Roth) for 15–30 min at RT. Fluorescent signals were quantitatively captured using the Odyssey DLx Infrared Imaging System (LI-COR Biosciences) and the half-maximal inhibitory concentration (IC_50_) values were calculated using GraphPad software (version 7 or newer).

### In vivo pharmacology

All mouse experiments were approved by the relevant regulatory agency (State Office for Health and Social Affairs, Berlin, Germany; approval number: Reg 0010/19) and conducted in compliance with the German Animal Welfare Act. Animals were kept in a 12 h light/dark cycle at a housing temperature of 23 °C. Food and water were available ad libitum. Therapies were well tolerated and decreases in body weight did not exceed 10% of body weight at the start of treatment; no drug-related deaths were observed.

CT26 tumor cells were cultivated in RPMI 1640 with 2 mM L-glutamine (Gibco, Invitrogen GmbH) containing 10% fetal bovine serum (Gibco) at 37 °C and 5% CO_2_. Cell lines were maintained in culture for no longer than 6 months, and mycoplasma contamination was excluded by enzymatic test (MycoAlert, Lonza) prior to in vivo application. Tumor cells (2 × 10^6^) were transplanted subcutaneously into 6- to 8-week-old female BALB/c mice. Control mice were subjected to sham surgery without tumor cell transplantation. Blood and tumor samples were collected from untreated control and tumor-bearing mice on days 4 and 8 (*n* = 3 mice/group). The remaining tumor-bearing mice were randomized on day 8, by which time the tumor size had reached approximately 100 mm^3^, to the control groups V1 and V2 (*n* = 6 mice/group) and to the treatment groups REG(V1) and REG(V2) (*n* = 18 mice/group), and treatment was immediately started. REG was administered at 10 mg/kg daily by oral gavage (5 mL/kg) for up to 10 days. For interim analysis, blood and tumor tissue were collected from each group (*n* = 3 mice/group) after 7 days of treatment. For pharmacokinetic (PK)/pharmacodynamic (PD) relationship analysis, tissues were taken 24 h after the penultimate dose and 1, 3, 7, and 24 h after the ultimate dose. Tumor diameter was measured by caliper and body weight was determined at least twice weekly. Tumor volume was calculated using the formula 0.5 × *a* × *b*^2^, where *a* and *b* are the long and short diameters of the tumor, respectively.

For tissue collection, mice were killed at predetermined time points. Blood samples were collected after retrobulbar venous plexus puncture in MiniCollect tubes containing ethylenediaminetetraacetic acid (EDTA; Greiner Bio-One). Typically, 100 µL of blood was used per flow cytometry (FC) staining. The remaining material was centrifuged for 10 min at 3000 g at 4 °C and the plasma supernatant was snap-frozen for further analyses. Tumors were removed, and their weights determined, then divided into three pieces, one of which was snap-frozen, one formalin fixed and paraffin embedded, and one weighed and used for FC analyses.

### Flow cytometry

For tumors, single-cell suspensions were prepared from the fresh tumor pieces using a mouse Tumor Dissociation Kit (Miltenyi Biotec). The final cell pellets were resuspended in 1 mL FC buffer (PBS with 2 mM EDTA, 0.5% bovine serum albumin). Then, 100 µL of the tumor single-cell suspensions and the EDTA blood samples were stained by addition of the antibody cocktail and incubated for 10 min at 4 °C according to the manufacturer’s instructions. After antibody incubation, blood samples were incubated with 2–3 mL lysis buffer (0.17 M NH_4_Cl, 10 mM KHCO_3_, 0.1 mM EDTA) for 10–20 min at RT to lyse and remove red blood cells. Subsequently, samples were centrifuged at 300 g for 5 min at 4 °C and cells were washed with 2–3 mL FC buffer. Tumor samples were washed immediately after antibody incubation with 2–3 mL FC buffer without the lysis procedure and centrifuged at 300 g for 5 min at 4 °C. Cell pellets from blood and tumor samples were resuspended in 100 µL and 200 µL FC buffer, respectively. Next, 50 µL of blood and 70 µL of tumor cell suspensions were analyzed by FC using MACSQuant Analyzer 10 and MACSQuant Analyzer 16 (Miltenyi Biotec). FC data were analyzed with the FlowLogic software (Inivai Technologies distributed through Miltenyi Biotec; version 7.2.1) and gated as shown in Fig. S1 (see Additional file 2), whereby up to seven subpopulations were identified in the CD45^+^ fraction using antibodies against F4/80 and CD115. In blood, the analysis focused on the CD115^hi^F4/80^−^ population, henceforth referred to as CD115^hi^. In tumor samples, the analysis focused on the F4/80^hi^CD115^lo^ population, henceforth referred to as F4/80^hi^.

### Enzyme-linked immunosorbent assay (ELISA)

CCL2 was detected in plasma samples and tumor tissue using the U-Plex mouse MCP-1 ELISA Kit (Meso Scale Diagnostics) according to the manufacturer’s instructions. Measurements were performed at least in duplicate. For plasma, 12.5 µL was analyzed per animal.

For tumors, snap-frozen tissue was subjected to cryogenic grinding using a Mixer Mill MM 400 and stainless steel beads (7 mm in diameter; Retsch) and subsequently lysed in Tris Lysis Buffer (Meso Scale Diagnostics). The lysates were centrifuged at 25,000 g at 4 °C for 10 min, protein concentrations were determined using the 660 nm protein assay kit (Pierce), and 2–20 μg of lysate was used for CCL2 analysis. CCL2 concentrations were related to the amounts of tumor tissue taken for the lysis procedure.

### Statistical analysis

Unless otherwise specified, formal hypothesis testing was conducted at the 5% confidence level for each statistical method. Analyses and computations were performed using SAS 9.4 software, except for the PK/PD analysis in the CT26 study, which was carried out using R 3.6.1.

The repeated tumor volume caliper measurements were compared to evaluate the difference in the growth slopes between vehicle and treated groups. All measurements starting from day 8 to day 14 of the experiment were included. A covariance pattern model was applied, assuming linear growth for the log_10_-transformed values, which corresponds to exponential growth when using the original values, and an AR(1) correlation structure between measurements of the same animal (see Additional file 1. Statistical analysis) [26].

One-sided Welch’s *t*-tests were applied for comparison of the average logarithmic CCL2 concentrations and logarithmic CD115^hi^ cell percentages of CD45^+^ cells, respectively, in PB of sham-treated mice without tumors and tumor-bearing mice on days 4 and 8 of the experiment.

An overall comparison between treatment groups of the endpoints measured on CT26 and MC38 mice after they had been killed was performed using different statistical models. All available measurements starting from day 15 of the experiment (CT26 study) and from day 14 of the experiment (MC38 study) were used for the evaluation, except for intra-daily observations in the final 24 h.

Five analysis of variance (ANOVA) models were applied to analyze the log_10_-transformed values of the tumor weight in the CT26 study, the CCL2 levels in the tumor in the CT26 and MC38 studies, and the CCL2 levels in the PB in the CT26 and MC38 studies. The standard assumption of equal variances between treatment groups was applied for all endpoints, except for CCL2 levels in the tumor in the MC38 study, for which unequal variances between treatment groups were allowed. A negative binomial regression model was applied to evaluate percentages of CD115^hi^ and F4/80^hi^ cells within the CD45^+^ cell population in PB and tumor tissue, respectively. Notably, tumor CCL2 measurements are the result of the geometric average of two to four replicates obtained from various ELISA measurements.

A PK/PD analysis using values from 24 h after the penultimate REG application (considered as baseline) and the subsequent intra-daily measurements at 1, 3, 7, and 24 h after the last REG application was performed to assess the correlation of the endpoints and the REG concentration at the intra-daily level. Response ratios with confidence interval [27] as deviation from baseline were derived for all endpoints, as well as for REG concentration in tumors and plasma. No formal hypothesis testing was conducted.

## Results

### REG and its metabolites M-2 and M-5 potently inhibit CSF1R in RAW264.7 macrophages

**Fig. 1:**
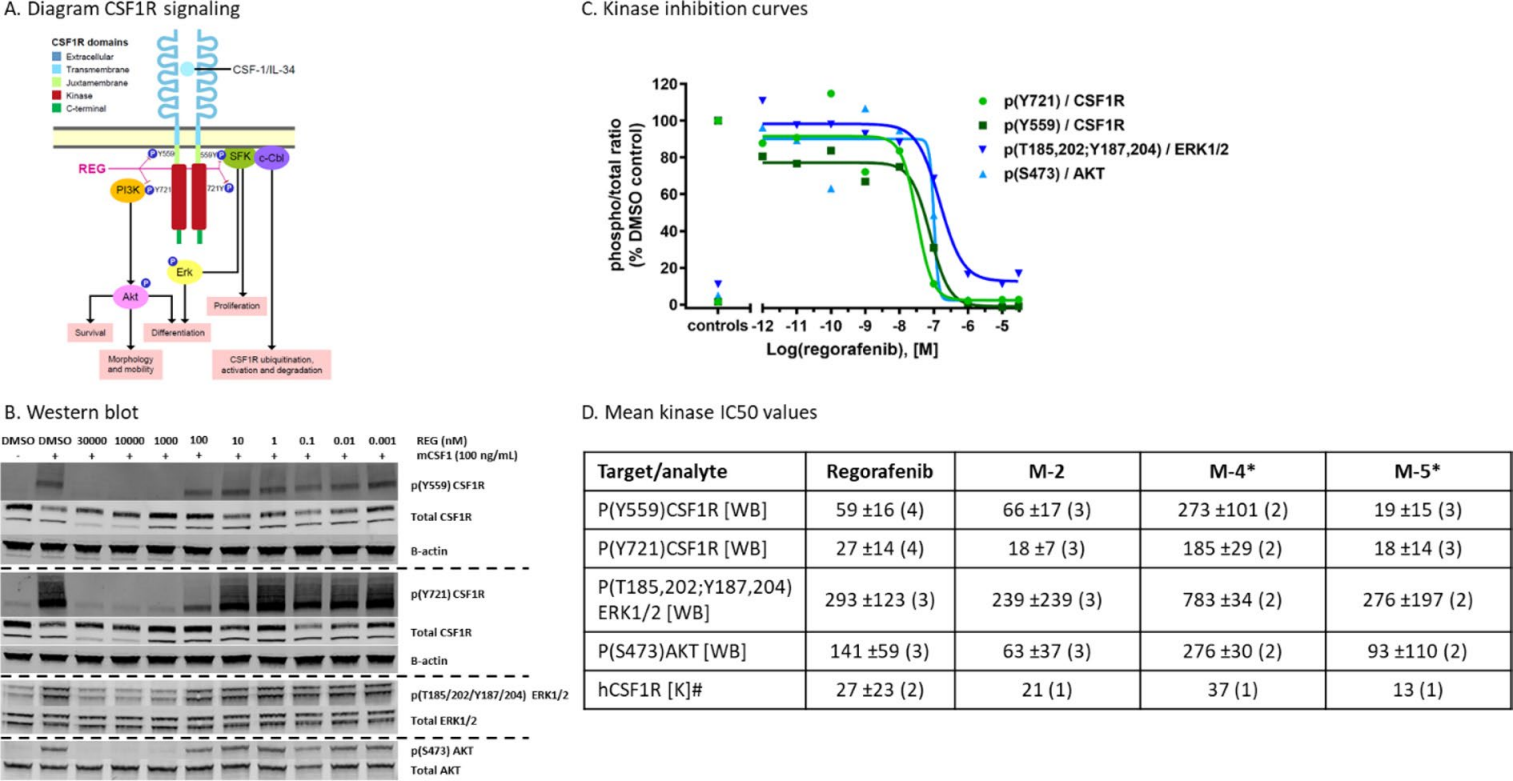
REG inhibits CSF1/CSF1R signaling in macrophages in vitro. **A** Schematic diagram of CSF1R signaling relating to this study. **B** Western blot analysis of total lysates (40 µg/lane) from mouse RAW264.7 cells treated as indicated. Tyrosine phosphorylation sites correspond to the murine CSF1R. The two phosphorylation sites were analyzed on separate blots together with the total CSF1R and β-actin as loading control. The pY721 blot was re-probed for total/pAKT and total/pERK was analyzed on a new blot. Fluorescent signals were captured densitometrically and used to determine IC_50_. The whole blot figure is shown in Fig. S3 (see Additional file 2). **C** Example IC_50_ determination using GraphPad software. DMSO without and with CSF1 stimulation was used as controls. **D** Cellular IC_50_ values of various kinases are given as means with standard deviation. The number of assay replicates is given in brackets. *IC_50_ for pCSF1R is derived directly from the densitometric values of the respective pCSF1R bands without relating to the signal of total CSF1R, which was destabilized by M-4 and M-5 (see Additional file 2: Fig. S2). ^#^Data for M-2 and M-5 taken from Zopf et al., 2016 [5]. IL, interleukin; K, competitive kinase binding assay; WB, western blot.

CSF1R is involved in the differentiation of TAMs from blood monocytes and is inhibited by REG and its metabolites M-2 and M-5 [5]. This finding was confirmed in vitro in murine RAW264.7 macrophages. REG, M-2, M-4, and M-5 potently inhibited CSF1R with cellular IC_50_ values between 18 and 273 nM across the two tested pY559 and pY721 phosphorylation sites, and these values correlated well with the biochemical values (Fig. 1A–D; see Additional file 2: Fig. S2). pY559 is described as being involved in receptor ubiquitination and degradation [28], and its inhibition was found to be consistently associated with concomitant reduction of ubiquitinated receptor (Fig. 1B). The activation of the intracellular kinases ERK and AKT, which mediate CSF1R signaling, was also inhibited by all three compounds, with IC_50_ in the range of 239–783 nM and 63–276 nM, respectively [29] (Fig. 1A–D). This is considered to be a result of CSF1/CSF1R signaling blockade because there was no significant biochemical inhibition of ERK and AKT by any of these compounds [4, 5]. The shutdown of the entire CSF1/CSF1R signaling cascade in macrophages by REG could explain the previously observed TAM effects.

### REG inhibits the subcutaneous growth of CT26 CRC tumors in a dose-dependent manner

**Fig. 2:**
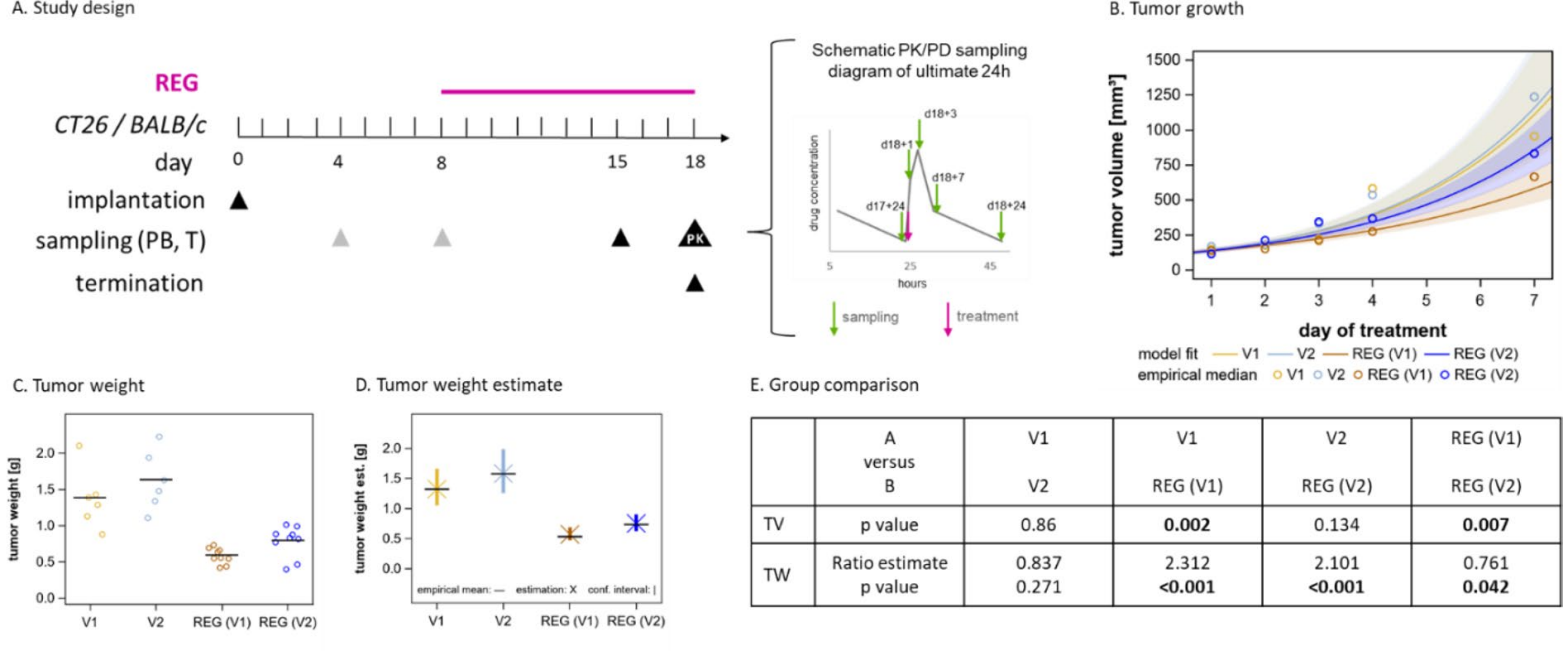
REG inhibits the growth of subcutaneous CT26 tumors in mice. **A** Schematic diagram of the study design including sampling details after the last REG dose. Gray triangles indicate sampling of untreated mice, black triangles of treated mice. **B** Fitted tumor growth curves and confidence areas based on tumor volume determinations within the exponential growth phase after treatment start. The untreated control was not included because the early sampling had an influence on the growth estimation. **C** Resected tumor weights after study termination. Tumors of the REG groups collected for PK analysis 1, 3, and 7 h after the last dose are omitted. Horizontal black lines represent mean values. V1 and V2 *n* = 6 tumors/group; REG(V1) and REG(V2) *n* = 9 tumors/group. **D** Tumor weight estimates determined as described in the statistical section of the methods. **E** Statistical significances derived from tumor growth model and tumor weight estimations. Significant *P*-values are indicated in bold. TV, tumor volume; TW, tumor weight.

To further investigate possible CSF1R-mediated immunomodulatory effects of REG in vivo, we first evaluated the effect of 10 mg/kg/day REG, which corresponds to the maximal clinical dose of 160 mg REG per day, on the subcutaneous growth of CT26 tumors in mice (Fig. 2A) [5, 30]. To rule out potential secondary vehicle effects on the immune system, REG was administered in a second vehicle (V2) in addition to the standard vehicle (V1). Pharmacokinetically, REG exposure, in terms of area under the curve during 24 h at steady state (AUC_(0–24)ss_) and maximum concentration (C_max_), was lower (1.5–2-fold) in both plasma and tumor tissue after administration in V2 compared with its administration in V1 (see Additional file 2: Fig. S4). Besides underlining the importance of PK analyses to assess vehicle-dependent drug exposures, this provided the possibility to evaluate dose-dependent effects. Tumor weight was significantly reduced with REG(V1) and REG(V2) vs vehicle control; tumor volume was significantly reduced with REG(V1) only vs vehicle control (Fig. 2B–E; see Additional file 2: Fig. S5). REG(V1) was more effective than REG(V2), which correlates with the higher exposure of REG in REG(V1) vs REG(V2) and demonstrates the dose-dependent antitumor activity of REG. The vehicle groups V1 and V2 were not significantly different.

### CT26 tumors increase the number of CD115^hi^ monocytes and the concentration of CCL2 in PB

**Fig. 3:**
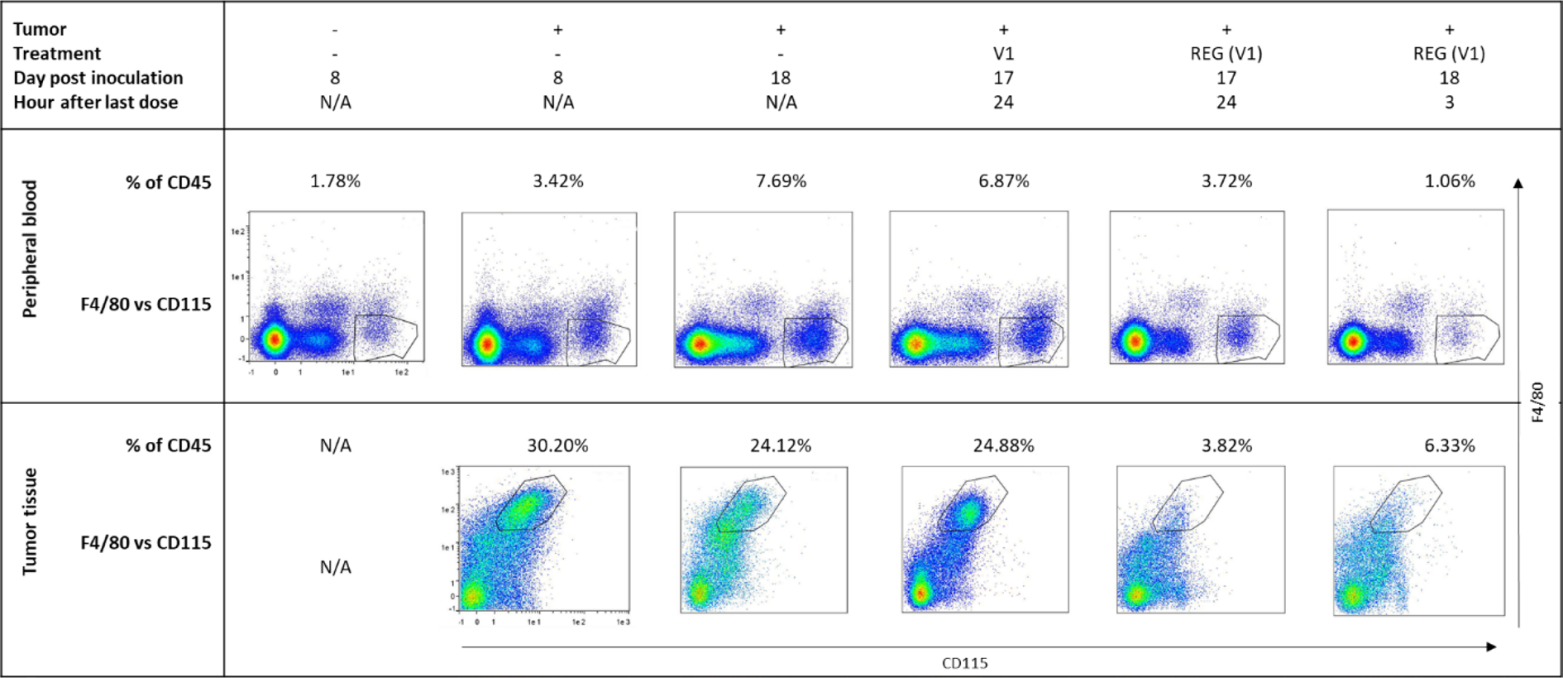
REG reduces CD115^hi^ cells in PB and F4/80^hi^ cells in tumors. Event analysis results are exemplified by selected single samples from the indicated groups. Regions of interest for CD115^hi^ cells in PB and F4/80^hi^ cells in tumor tissue are framed. N/A, not applicable.

Protumorigenic TAMs are derived from blood monocytes, which are attracted by cytokines released by tumors, such as CCL2 [31]. We analyzed whether CT26 tumors affected monocytes and CCL2 levels in PB using FC gating for the REG target CD115 (see Additional file 2: Fig. S1A) and ELISA, respectively. Both the concentration of CCL2 and the percentage of CD115^hi^ cells were significantly higher in mice with tumors than in sham-treated control mice without tumors 8 days after the start of the experiment, but not after 4 days (Fig. 3; see Additional file 2: Fig. S6). This indicates that CT26 tumors may attract monocytes by secretion of CCL2 and suggests that surgery did not have an effect. As REG inhibits CSF1R, we then examined its effects on monocytes and CCL2.

### REG reduces the number of CD115^hi^ monocytes but had no effect on CCL2 levels in PB

**Fig. 4:**
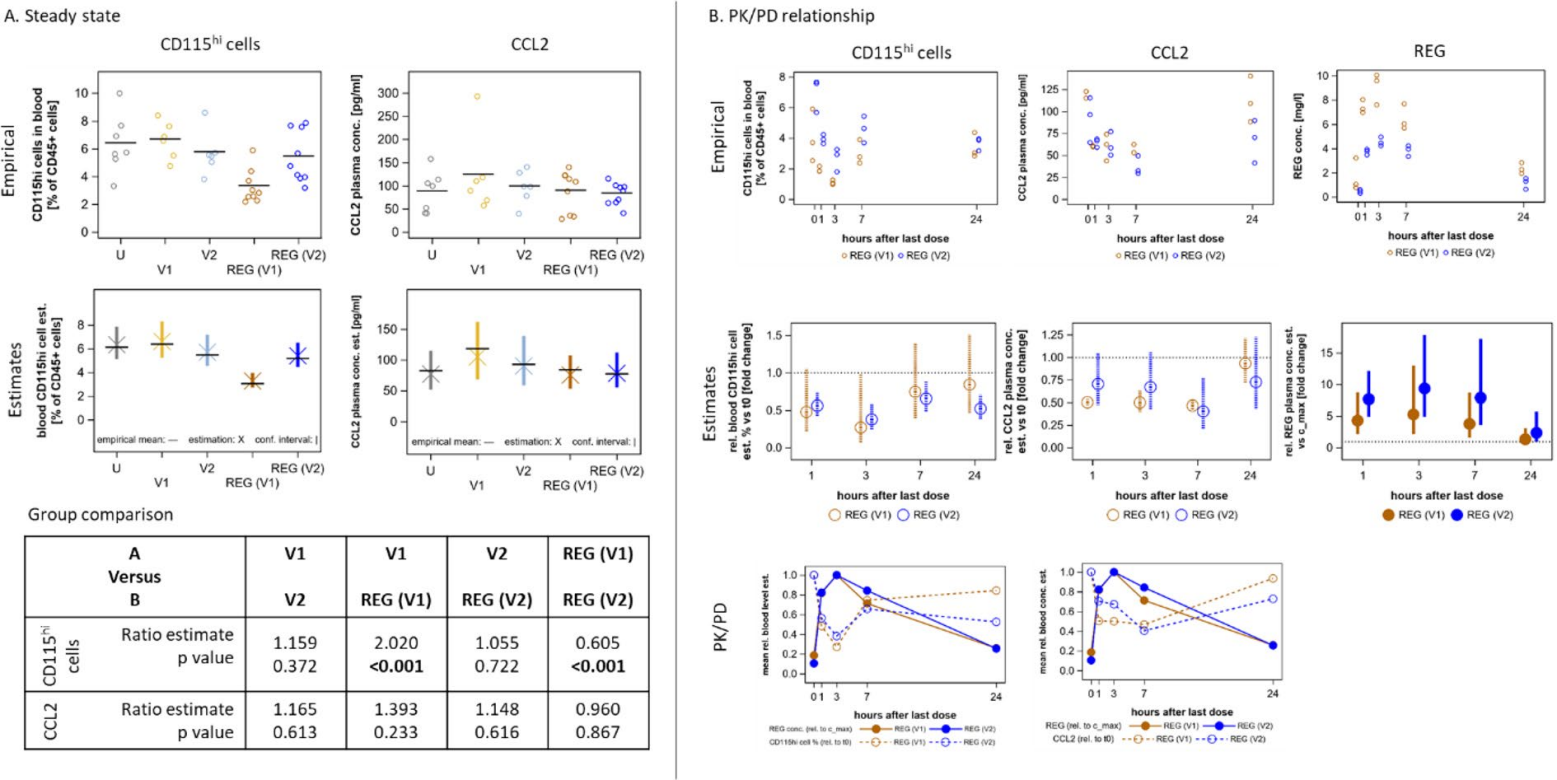
REG differentially affects the number of CD115^hi^ cells and the CCL2 levels in PB. Blood samples were collected according to the study design (Fig. 2A) and analyzed for CD115^hi^ cells by FC, for CCL2 by ELISA, and for REG by liquid chromatography–tandem mass spectrometry. Empirical data (emp) were analyzed as described in the statistical methods section and the resulting estimates (est) are shown below the corresponding empirical graphs.** A** Steady-state analysis. Samples of the REG groups collected for PK analysis 1, 3, and 7 h after the last dose are omitted. V1 and V2 *n* = 6 animals/group; REG(V1) and REG(V2) *n* = 9 animals/group. Horizontal black lines represent mean values. Results of statistical tests on mean ratios for group comparison are tabulated. Significant *P*-values are indicated in bold.** B** PK/PD relationship analysis (*n* = 3 animals/group). Empirical data were analyzed as described in the statistical methods section and the resulting mean estimates with confidence interval (vertical bars) are presented below the corresponding empirical graphs as fold change relative to t_0_ for CD115^hi^ and CCL2 and relative to C_max_ for REG. The horizontal dotted black line represents the baseline. Confidence intervals completely below or above the baseline indicate significant changes. The relationship of REG with CD115^hi^ and with CCL2 (PK/PD) is depicted based on the respective relative mean estimates.

CD115^hi^ monocytes were analyzed by FC in PB samples taken 24 h after the last dose of 7, 10, or 11 daily treatments with REG to assess the steady-state treatment effect (Fig. 2A). REG(V1) significantly reduced the number of CD115^hi^ cells in PB vs V1 by about half (Fig. 3, Fig. 4A [CD115^hi^ cells]). No significant effect was observed for REG(V2) in comparison with V2, which is in line with the lower REG exposure in REG(V2) and indicates that this effect may occur only at a higher REG exposure. V1 and V2 did not differ from samples of untreated mice, suggesting that these two vehicles did not affect these cells. REG did not change CCL2 levels in corresponding plasma samples in this steady-state analysis vs vehicle or untreated samples (Fig. 4A [CCL2]). Similarly, REG reduced the number of CD115^hi^ cells in the blood of C57BL/6 mice with subcutaneous MC38 CRC tumors without affecting the CCL2 levels (see Additional file 2: Fig. S7A and C), indicating that this effect is not model specific.

### REG transiently lowers the number of CD115^hi^ monocytes and the levels of CCL2 in PB within 24 h after the last dose, inverse to its concentration

To evaluate PK/PD relationships, we analyzed blood samples taken at various time points within 24 h after the last REG dose (Fig. 2A). Both CD115^hi^ monocytes and CCL2 were significantly reduced by REG(V1) and REG(V2) over time. This reduction became evident 1 h after the last dose and persisted until 3 h and 7 h, respectively, before levels returned close to steady-state baseline by 24 h (Fig. 3 [Peripheral blood], Fig. 4B). The highest concentration of REG (C_max_) in corresponding plasma samples occurred by 3 h before returning to baseline (Fig. 4B [REG]). A negative correlation was observed comparing the time curves of the relative concentration estimates between REG and both PD measures, CD115^hi^ cells and CCL2 (Fig. 4B [PK/PD]). This strongly indicates the involvement of REG, and thereby establishes a clear PK/PD relationship.

### REG significantly reduces the number of intratumoral F4/80^hi^ macrophages and increases CCL2 levels

**Fig. 5:**
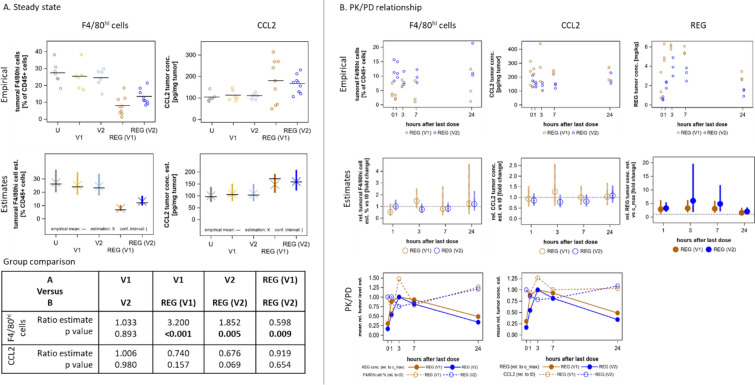
REG differentially affects the number of F4/80^hi^ cells and the CCL2 levels in tumor tissue. Tumor samples were collected according to the study design (Fig. 2A) and analyzed for F4/80^hi^ cells by FC, for CCL2 by ELISA, and for REG by liquid chromatography–tandem mass spectrometry. Empirical data (emp) were analyzed as described in the statistical methods section and the resulting estimates (est) are shown below the corresponding empirical graphs.** A** Steady-state analysis. Samples of the REG groups collected for PK analysis 1, 3, and 7 h after the last dose are omitted. V1 and V2 *n* = 6 animals/group; REG(V1) and REG(V2) *n* = 9 animals/group. Horizontal black lines represent mean values. Results of statistical tests on mean ratios for group comparison are tabulated. Significant *P*-values are indicated in bold.** B** PK/PD relationship analysis (*n* = 3 animals/group). Empirical data were statistically analyzed as described in the respective methods sections and the resulting mean estimates with confidence interval (vertical bars) are presented below the respective empirical data as fold change relative to t_0_ for F4/80^hi^ and CCL2 and relative to C_max_ for REG. The horizontal dotted black line represents the baseline. Confidence intervals completely below or above the baseline indicate significant changes. The relationship of REG with F4/80^hi^ and with CCL2 (PK/PD) is depicted based on the respective relative mean.

Fresh tumor tissue was dissociated and analyzed by FC using the same gating strategy as applied for the PB cells (see Additional file 2: Fig. S1B). In untreated animals, a new intratumoral immune cell population, not found in the blood, was identified, which expressed high levels of F4/80 and low levels of CD115 (Fig. 3 [Tumor tissue]; see Additional file 2: Fig. S1B). These F4/80^hi^ cells, considered to reflect TAMs, amounted to approximately 25% of the CD45^+^ fraction in untreated mice or vehicle controls. They were detected on day 8 of tumor growth and remained at a constant level until the end of the study (Fig. 3 [Tumor tissue]). In contrast to the blood, only very small amounts (< 0.5%) of CD45^+^CD115^hi^ cells were detected in the tumor. In the steady-state analysis, REG(V1) reduced the number of F4/80^hi^ cells significantly and dose dependently about threefold, and REG(V2) about twofold, vs V1 and V2, respectively. As expected from their different exposures, REG(V1) was more effective than REG(V2). The vehicle groups did not differ from untreated animals (Fig. 5A). When the CCL2 concentrations were determined in corresponding tumor lysates, a trend toward higher levels was found in the tumors after treatment with REG vs vehicle controls (Fig. 5A [CCL2]). Comparable results were also obtained with MC38 tumors. REG significantly reduced the F4/80^hi^ population, which also appeared in these tumors, by more than twofold, even at a dosage of 5 mg/kg/day, vs vehicle (see Additional file 2: Fig. S7B and C). REG also dose dependently increased CCL2 concentrations in MC38 tumors (see Additional file 2: Fig. S7B and C).

### No PK/PD relationships between REG and intratumoral F4/80^hi^ macrophages or CCL2 occur in tumor tissue

For PK/PD relationships in the tumor, samples from the same time points as for blood were analyzed (Fig. 2A). Marginal changes were observed for both PD markers, which statistically did not deviate significantly from baseline levels (Fig. 5B). When the concentration–time curves between REG and F4/80^hi^ cells and CCL2 were compared, an overlapping profile was observed for both PD markers (Fig. 5B [PK/PD]). At REG’s C_max_, both markers exceeded baseline in the REG(V1) group, which had a higher REG exposure; they were below baseline in the REG(V2) group, in which the REG exposure was only half of that in the REG(V1) group. This observation relates to previous findings [11, 17] and supports the suggestion that REG may exert its immunomodulatory activity at an optimal biological dose.

### REG reduces selective subpopulations of TAMs

REG treatment did not deplete tumor tissue entirely from TAMs (Fig. 5A [F4/80^hi^]). We applied quantitative real-time PCR (qRT-PCR) to characterize the remaining TAM fraction. We selected various TAM-associated genes, including candidate genes specific for recently identified subpopulations [32], and compared REG(V1) with V1 tumor samples. REG significantly reduced the transcript frequencies of various genes, such as *Adgre1* (F4/80) and *CSF1R* (see Additional file 2: Fig. S8), which is in line with our FC data, as well as Mrc1 (CD206), a marker for protumorigenic M2-TAMs, previously shown by immunofluorescence staining to be reduced by REG in orthotopic CT26 tumors [12]. Furthermore, the frequencies of Maf and Mgl2, markers of TAM subpopulations considered to be associated with sensitivity to CSF1R inhibitors, were also reduced, whereas the frequencies of MafB, VEGFA, and Hilpda, indicators of resistance to CSF1R inhibitors [32], as well as Arg1 and Nos2, were unchanged. The fact that REG does not reduce Nos2, a M1-TAM marker, in contrast to Mrc1, is an indicator of an increased M1:M2 ratio impeding tumor growth.

## Discussion

**Fig. 6:**
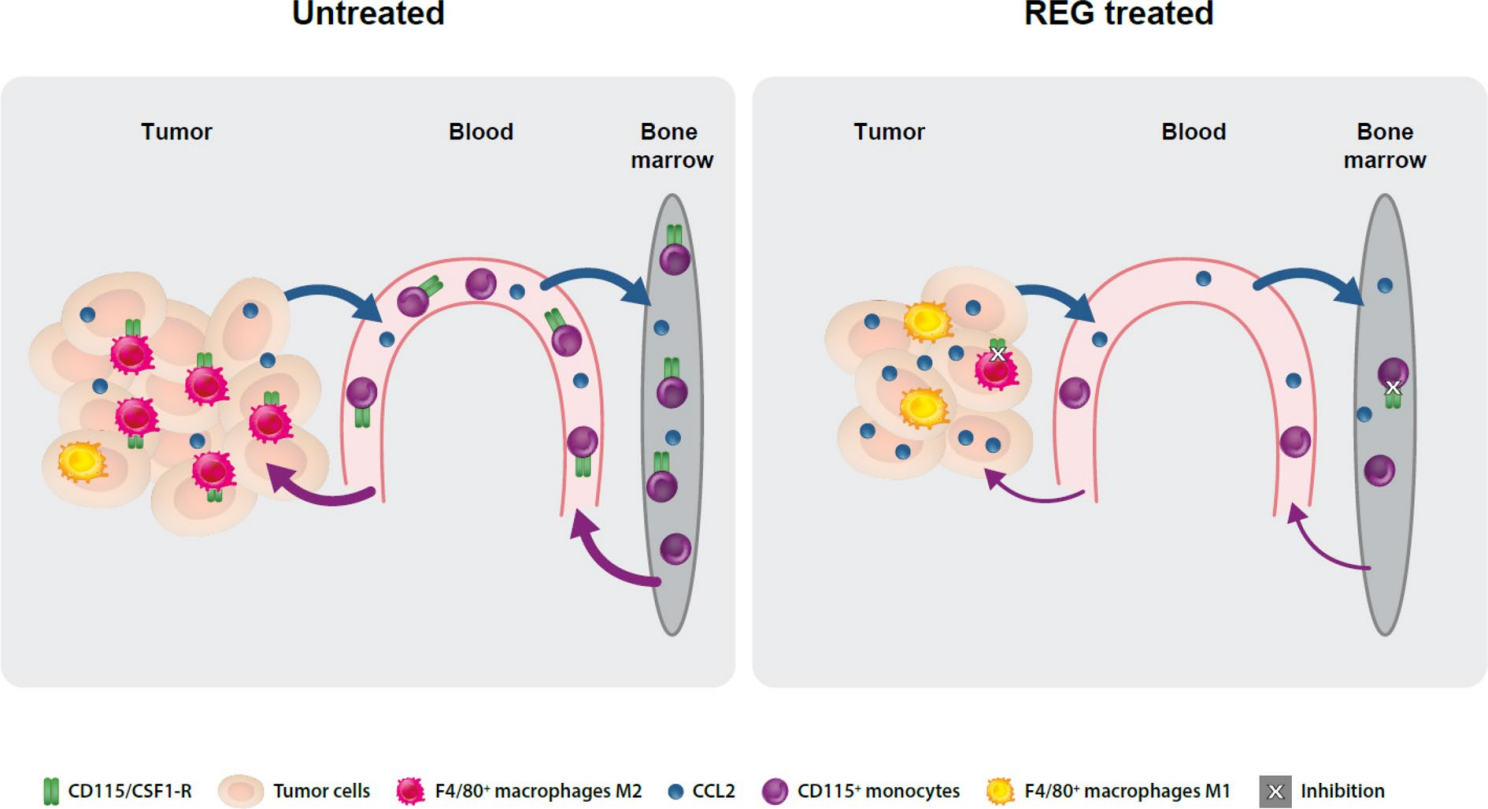
Immunomodulatory effects of regorafenib via inhibition of CSF1R. Untreated CRC tumors release CCL2 into the circulation, which induces an increase of CD115^hi^ immune cells in the blood, presumably mostly monocytes mobilized from bone marrow precursors. This is accompanied by a rise in F4/80^hi^ TAMs, which also express CD115, although at a lower level compared with the CD115^hi^ cells in the blood, and which mediates their survival, proliferation, and differentiation into the protumorigenic M2 subtype. In tumor-bearing mice treated with REG, inhibition of CSF1R results in a concerted and significant reduction in the number of CD115^hi^ cells in the blood and F4/80^hi^ cells in the tumor. Residual TAMs by default become the antitumorigenic M1 subtype. This contributes to tumor growth inhibition, together with other effects mediated by the inhibition of multiple other kinases by REG. Complete tumor remission by REG may be prevented by upregulation of tumor-trophic CCL2.

In this study, we found that after prolonged treatment, REG significantly and persistently reduced CD115^hi^ monocytes in PB and in parallel F4/80^hi^ macrophages in tumors in two different CRC models, by inhibition of CSF1R. The reduction of tumor-growth-promoting F4/80^hi^ M2 macrophages in favor of antitumorigenic M1 macrophages relieves the immunosuppressive environment and contributes to tumor growth inhibition by REG. However, we also observed a persistent increase in CCL2 in CRC tumors, but not in PB, which may protect tumors from complete elimination by REG and eventually cause resistance to REG treatment (Fig. 6).

Untreated CRC tumors release CCL2 into the circulation, which is accompanied by an increase of CD115^hi^ monocytes in PB and strongly suggests that CD115^hi^ monocytes are mobilized by CCL2, most likely from the bone marrow, thereby confirming previous findings [13]. By day 8 after tumor cell inoculation, blood levels of CCL2 and CD115^hi^ cells were significantly higher than in sham-treated mice without tumors, indicating that these effects are tumor specific. CCL2 may be derived directly from CT26 and MC38 tumor cells, as observed in in vitro cultures [33–35], although this was not seen for CT26 cells by others [36], or induced in vivo under hypoxic conditions in tumor cells or other cells of the tumor microenvironment [37, 38]. We cannot exclude that other chemokines such as CCL5, which can mobilize CD115^hi^ monocytes, are also released [39]. By day 8, tumors contain a large population of F4/80^hi^ macrophages, which are presumably derived at least partially from the activated CD115^hi^ cells in the PB. They express CD115, although at a lower level than CD115^hi^ cells, which are found in the PB. When activated by CSF1, they differentiate into M2-type macrophages accompanied by the CSF1-regulated expression of F4/80 [40].

In REG-treated mice, CD115^hi^ and F4/80^hi^ cells are significantly reduced, which is most likely mediated by inhibition of CSF1R for the following reasons. Similar reductions of blood monocytes and TAMs were observed in CSF1R knock-out mice or mice treated with selective CSF1R inhibitors [21, 41, 42]. In vitro, REG potently inhibited murine CSF1R in RAW264.7 macrophages with an IC_50_ of approximately 30 nM. The cumulative unbound drug concentration of REG, M-2, M-4, and M-5 estimated from their maximal tumor concentrations is well above the in vitro IC_50_ both in plasma and in tumor tissue, which should enable CSF1R inhibition in vivo. Notably, in contrast to humans, significant amounts of M-4 accumulate during the biotransformation of REG in mice. M-4 has similar pharmacologic activity to REG, M-2, and M-5, and may cumulatively add to the pharmacologic activity of REG. In a competition binding assay, M-4 inhibits the reference kinase VEGFR2, with an IC_50_ of 37 nM compared with 14 nM for REG. Similarly, it exerts high binding to mouse plasma, resulting in an unbound fraction of 0.332%, comparable to REG, M-2, and M-5 [5]. Although we tried various approaches, we could not reproducibly demonstrate the reduction of phospho-CSF1R by REG in vivo, despite the detection of total CSF1R. This may be due to the high instability of phospho-CSF1R or to its levels being below the detection limits of our assays. Finally, a negative PK/PD relationship between CD115^hi^ cells and REG was observed in the blood.

TAMs became a therapeutic target because their elevated numbers have been correlated with poor prognosis in patients with different cancers, and their inactivation is considered to improve clinical outcomes [39, 43]. However, in CRC the situation is less clear because favorable effects of TAMs have also been reported [44], and it may be necessary to differentiate between primary and metastatic tumors and between different macrophage subtypes, which were recently described [45]. REG did not fully eliminate the intratumoral F4/80^hi^ cells. It appears to selectively reduce those that are sensitive to an inhibitory anti-CSF1R antibody and not those that are resistant to the antibody. A resistant subtype expressing VEGF-A was associated with the tumor vasculature and speculated to promote angiogenesis, thereby hampering antitumor activity. By inhibiting VEGFRs, REG may overcome the effects of this resistant macrophage subtype. REG had no significant impact on antitumorigenic M1 macrophages as their marker Nos2 remained unchanged in the qRT-PCR analysis, in contrast to the M2 macrophage marker CD206/Mrc1, which was strongly reduced. This could lead to an improved M1:M2 ratio, confirming previous immunofluorescence data in orthotopic CT26 tumors [12], and favorably affect antitumor activity.

REG significantly inhibited tumor growth vs vehicle but did not induce partial or complete remission. Seemingly, reducing protumorigenic TAMs and inhibiting VEGFR2 signaling, which not only blocks angiogenesis but also affects other immune cells such as regulatory and cytotoxic T cells, are insufficient to eliminate the tumor. Notably, this includes other receptor tyrosine kinases such as VEGFR1 and cKIT, which are also involved in immunological processes and inhibited by REG [46]*.* We found that sustained REG treatment leads to an increase of intratumoral CCL2, a potential counteracting mechanism. CCL2 was shown to protect tumor cells and may even induce resistance, as shown for doce-taxel in lung cancer cells and for sorafenib in HCC [47, 48]. Inhibitors of the CCL2/CCR2 signaling pathway have been tested clinically [49] and may be beneficial in combination with REG. In support of this, curcumin, among other drugs, was shown to reduce CCL2 [49]; a different study observed a synergistic effect of REG and curcumin in preclinical CRC models [50].

## Conclusions

Our data may be of clinical relevance. Reduction of CD14^dim^CD16^+^ monocytes was shown in the PB of patients with glioblastoma treated with the highly selective CSF1R inhibitor PLX3397 [21] and of patients with diffuse-type giant cell tumor treated with the anti-CSF1R antibody RG7155 [42]. If this also occurs in patients treated with REG, it could serve as an easy blood-based marker to monitor the activity of REG and support dose optimization for combination therapies or for personalized monotherapy given the highly variable PK of REG in patients [30, 51]. Elevated baseline plasma levels of CCL2 and CCL5 were associated with REG nonresponders in a small clinical study in patients with CRC [16]. Our data suggest that it might be worth determining the levels of CCL2 in tumor biopsies post-REG treatment to evaluate resistance mechanisms and provide indications for subsequent combination therapies.

## Supplementary Information

**Additional file 1: Table S1.** Antibody list. **Table S2.** qRT-PCR probes. Supplementary methods. In vivo pharmacology (MC38 study), PK, qRT-PCR, Statistical analysis, References.

**Additional file 2: Supplementary figures. Figure S1.** FC gating schemes for the detection of CD115- and/or F4/80-expressing immune cells in PB and CT26 tumor tissue. **Figure S2.** REG metabolites M-2, M-4, and M-5 inhibit CSF1/CSF1R signaling in macrophages in vitro. **Figure S3.** Entire western blots corresponding to the cropped regions depicted in Fig. 1B. **Figure S4.** PK parameters of REG and its metabolites M-2, M-4, and M-5 in **A** plasma and **B** CT26 tumor tissue from BALB/c mice. **Figure S5.** REG inhibition of CT26 tumor growth, represented by individual tumor growth curves. **Figure S6.** CT26 tumors induce elevation of CD115^hi^ cells and CCL2 in PB. **Figure S7.** Effects of REG on CD115^hi^ cells, F4/80^hi^ cells, and CCL2 in C57BL/6 mice with MC38 CRC tumors at steady state. **Figure S8.** Effects of REG on macrophage subpopulations by qRT-PCR analysis of selected marker genes.

## Data Availability

All methods and materials used are described in the manuscript and data can be obtained from the corresponding author upon request.

## Abbreviations

AKT: Protein kinase B
ANOVA: Analysis of variance
AUC_(0–24)ss_: Area under the curve during 24 h at steady state
CCL2: Chemokine (C–C motif) ligand 2
CCR: C–C chemokine receptor
C_max_: Maximum concentration
CRC: Colorectal cancer
CSF1: Colony-stimulating factor 1
CSF1R: Colony-stimulating factor 1 receptor
CXCL10: C-X-C motif chemokine ligand 10
DMSO: Dimethylsulfoxide
EDTA: Ethylenediaminetetraacetic acid
ELISA: Enzyme-linked immunosorbent assay
emp: Empirical
est: Estimate/s
ERK: Extracellular-signal regulated kinase
FC: Flow cytometry
GIST: Gastrointestinal stromal tumor
HCC: Hepatocellular carcinoma
IC_50_: Half-maximal inhibitory concentration
IL-34: Interleukin 34
MCP-1: Monocyte chemoattractant protein-1
Nos2: Nitric oxide synthase 2
PB: Peripheral blood
PBS: Phosphate-buffered saline
PCR: Polymerase chain reaction
pCSF1R: PhosphoCSF1R
PD: Pharmacodynamic
PK: Pharmacokinetic
qRT-PCR: Quantitative real-time PCR
REG: Regorafenib
RT: Room temperature
TAM: Tumor-associated macrophage
V: Vehicle
VEGF: Vascular endothelial growth factor
VEGFR: Vascular endothelial growth factor receptor
