# Correction: Effects of regorafenib on the mononuclear/phagocyte system and how these contribute to the inhibition of colorectal tumors in mice

**DOI:** 10.1186/s40001-023-01150-2

**Published:** 2023-06-24

**Authors:** Sylvia Grünewald, Maria Stecklum, Manuel Rizzo, Jonathan Rathjens, Lukas Fiebig, Dieter Zopf

**Affiliations:** 1 Bayer AG, Berlin, Germany; 2 Present Address: Nuvisan ICB GmbH, Berlin, Germany; 3 EPO Berlin-Buch GmbH, Berlin, Germany; 4 Chrestos Concept GmbH & Co. KG, Essen, Germany

**Correction: European Journal of Medical Research (2023) 28:147** 10.1186/s40001-023-01099-2

In the original publication of the article, the order of affiliations was incorrect. The corrected order of affiliations is given in this Correction article. The abbreviations “conc Concentration” and “Y Tyrosine” were included inadvertently though they are not abbreviated in text. The abbreviations of Concentration and Tyrosine were removed. The original article has been corrected.
