# Supplementary material for: Effects of regorafenib on the mononuclear/phagocyte system and how these contribute to the inhibition of colorectal tumors in mice
Source: Eur J Med Res. 2023 Apr 3;28:147. doi: 10.1186/s40001-023-01099-2 (PMC10069031; doi:10.1186/s40001-023-01099-2)
Supplement: Supplementary file 1 — Additional file 1: Table S1. Antibody list. Table S2. qRT-PCR probes. Supplementary methods. In vivo pharmacology (MC38 study), PK, qRT-PCR, Statistical analysis, References. [file 40001_2023_1099_MOESM1_ESM.docx]

# Additional file 1

## Table S1. Antibody list

APC, allophycocyanin; BD, Becton Dickinson; CST, Cell Signaling Technology; FC, flow cytometry; PE, phycoerythrin; PerCP, peridinin–chlorophyll protein complex; WB, western blot

## Table S2. qRT-PCR probes

Probes were obtained from Thermo Fisher Scientific

**Supplementary methods**

## In vivo pharmacology (MC38 study)

In vivo passaging was initiated by transplanting fragments of cryo-preserved MC38 tumors of early passages (<p5) subcutaneously into 6–8-week-old female C57BL/6 mice. Tumors were grown to a size of about 1000 mm^3^, excised, cut into fragments edging approximately 2 mm, and used for further passaging in vivo for a maximum of 10 passages. Necrotic areas were removed. For this study, 2 mm MC38 tumor fragments derived from passage 7 tumors were transplanted subcutaneously into 6–8-week-old female C57BL/6 mice. On day 7, when tumors had reached volumes of approximately 100 mm³, tumor-bearing mice were randomized to the untreated group U (*n* = 6), the vehicle control group V1 (*n* = 6), and treatment groups REG(5) (*n* = 6) and REG(10) (*n* = 8). Treatment was started the same day and REG was administered daily at 5 mg/kg (REG(5)) or 10 mg/kg (REG(10)) by oral gavage for up to 15 days. Blood and tumor samples were collected 24 h after 7 (all groups but U), 10 (groups U and V1), and 15 (all groups but V1) treatments. The REG(5) study was performed separately and independent from the REG(10) study with the following modifications: REG was injected at 10 mL/kg and no sample was collected at 24 h after the penultimate dose.

## PK

REG and its metabolites M-2, M-4, and M-5 were analyzed in plasma and tumor samples using validated bioanalytical liquid chromatography-tandem mass spectrometry methods according to an experimental protocol described earlier [1]. PK parameters, namely
AUC_(0–24)ss_ and C_max_, were calculated from plasma and tumor concentrations using a noncompartmental analysis.

## qRT-PCR

For mRNA expression analysis, snap-frozen tumor samples (20–30 mg, *n* = 3 tumor samples/group) were homogenized in 400 µL RLT buffer using the TissueLyser (Qiagen), followed by total RNA extraction using the RNeasy Plus Mini Kit (Qiagen) and cDNA synthesis using the SuperScript III First-Strand Synthesis SuperMix for qRT-PCR (Invitrogen GmbH), following the manufacturer’s instructions. RNA concentrations were determined spectrophotometrically at 260 nm using a Nanodrop 2000 instrument (Thermo Fisher Scientific) and its quality was assessed by the ratios of 260 nm to 280 nm and 230 nm, respectively. Gene expression analysis was performed by qRT-PCR in a 384-well plate (Micro-Amp Optical 384-Well Reaction Plate, Applied Biosystems, Thermo Fisher Scientific) using a ViiA 7 Real-Time PCR System (Applied Biosystems). The TaqMan PCR reaction was prepared in 10 μL containing 10 ng cDNA and the primer and probes for the respective target genes and for GAPDH (Table S2) using 2 × TaqMan Universal Master Mix (Thermo Fisher Scientific) following the manufacturer’s instructions. Relative mRNA expression was calculated using the ΔΔCt method and endogenous GAPDH mRNA expression as reference.

## Statistical analysis

The repeated-measurements covariance pattern model fitted to the longitudinal tumor volume data is specified as follows: let y*_ikt_* represent the logarithm (base 10) of the tumor volume, measured in animal *i*, which belongs to treatment group *k*, at time *t*. To represent the dependence of the tumor volume on time, distinguished by treatment group, the following model was assumed:

y*_ikt_ = α + β⋅t +* γ*_k_⋅t +* e*_ikt_*

with intercept *α*, overall slope *β*, group-specific slope γ*_k_*, and random error e*_ikt_*. The covariance structure of the latter between observations from the same animal was assumed to be *Cov(*e*_ikt_,* e*_iks_) =* σ*^2^⋅ρ^d(t,s)^*, with variance parameter *σ^2^>0*, correlation parameter *0<ρ<1*, and temporal distance *d(t,s)* between the measurements. No correlation was assumed between observations from different animals. Finally, the contrast *γ_k_-γ_l_* between growth slopes of treatment groups k and l was evaluated and the null-hypothesis *γ_k_-γ_l_ = 0* was tested, that is, that the average log-tumor volume growth rate is the same in both groups.

A two-sided *t*-test was conducted to evaluate the difference in transcript frequency, analyzed by qRT-PCR, for a selection of genes of interest from three CT26 tumors in each of the vehicle groups at 24 h after the penultimate treatment and in the REG(V1) group at 24 h after the penultimate and ultimate treatments.

**References**

1. Hafner FT, Werner D, Kaiser M. Determination of regorafenib (BAY 73-4506) and its major human metabolites BAY 75-7495 (M-2) and BAY 81-8752 (M-5) in human plasma by stable-isotope dilution liquid chromatography-tandem mass spectrometry. Bioanalysis. 2014;6:1923-37.
