# Supplementary material for: Effects of regorafenib on the mononuclear/phagocyte system and how these contribute to the inhibition of colorectal tumors in mice
Source: Eur J Med Res. 2023 Apr 3;28:147. doi: 10.1186/s40001-023-01099-2 (PMC10069031; doi:10.1186/s40001-023-01099-2)
Supplement: Supplementary file 2 — Additional file 2: Supplementary figures. Figure S1. FC gating schemes for the detection of CD115- and/or F4/80-expressing immune cells in PB and CT26 tumor tissue. Figure S2. REG metabolites M-2, M-4, and M-5 inhibit CSF1/CSF1R signaling in macrophages in vitro. Figure S3. Entire western blots corresponding to the cropped regions depicted in Fig. 1B. Figure S4. PK parameters of REG and its metabolites M-2, M-4, and M-5 in A plasma and B CT26 tumor tissue from BALB/c mice. Figure S5. REG inhibition of CT26 tumor growth, represented by individual tumor growth curves. Figure S6. CT26 tumors induce elevation of CD115hi cells and CCL2 in PB. Figure S7. Effects of REG on CD115hi cells, F4/80hi cells, and CCL2 in C57BL/6 mice with MC38 CRC tumors at steady state. Figure S8. Effects of REG on macrophage subpopulations by qRT-PCR analysis of selected marker genes. [file 40001_2023_1099_MOESM2_ESM.docx]

# Additional file 2

**Supplementary figures**


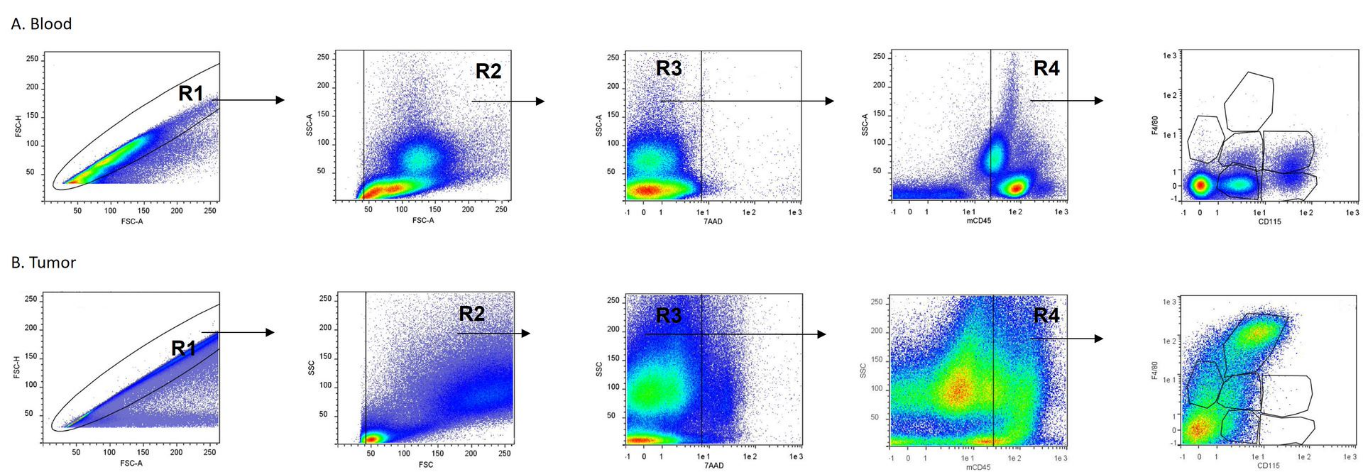


**Fig. S1** FC gating schemes for the detection of CD115- and/or F4/80-expressing immune cells in PB and CT26 tumor tissue

**
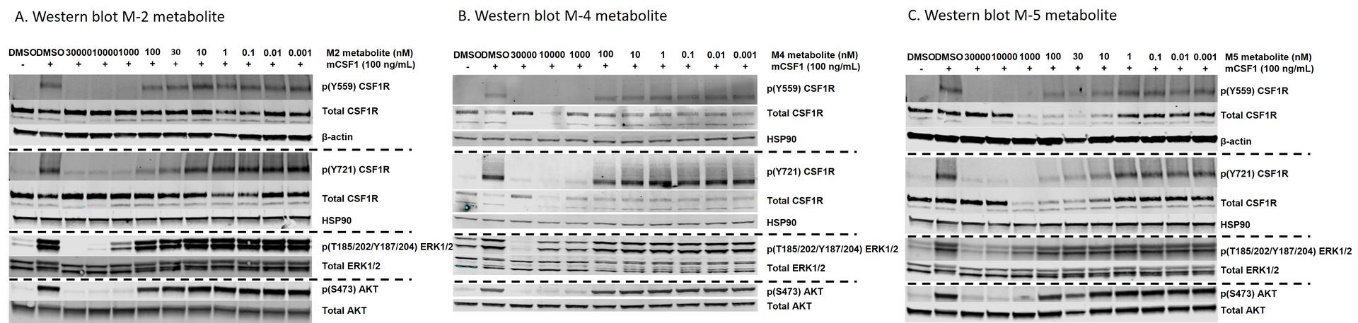
**

**Fig. S2** REG metabolites M-2, M-4, and M-5 inhibit CSF1/CSF1R signaling in macrophages in vitro

**
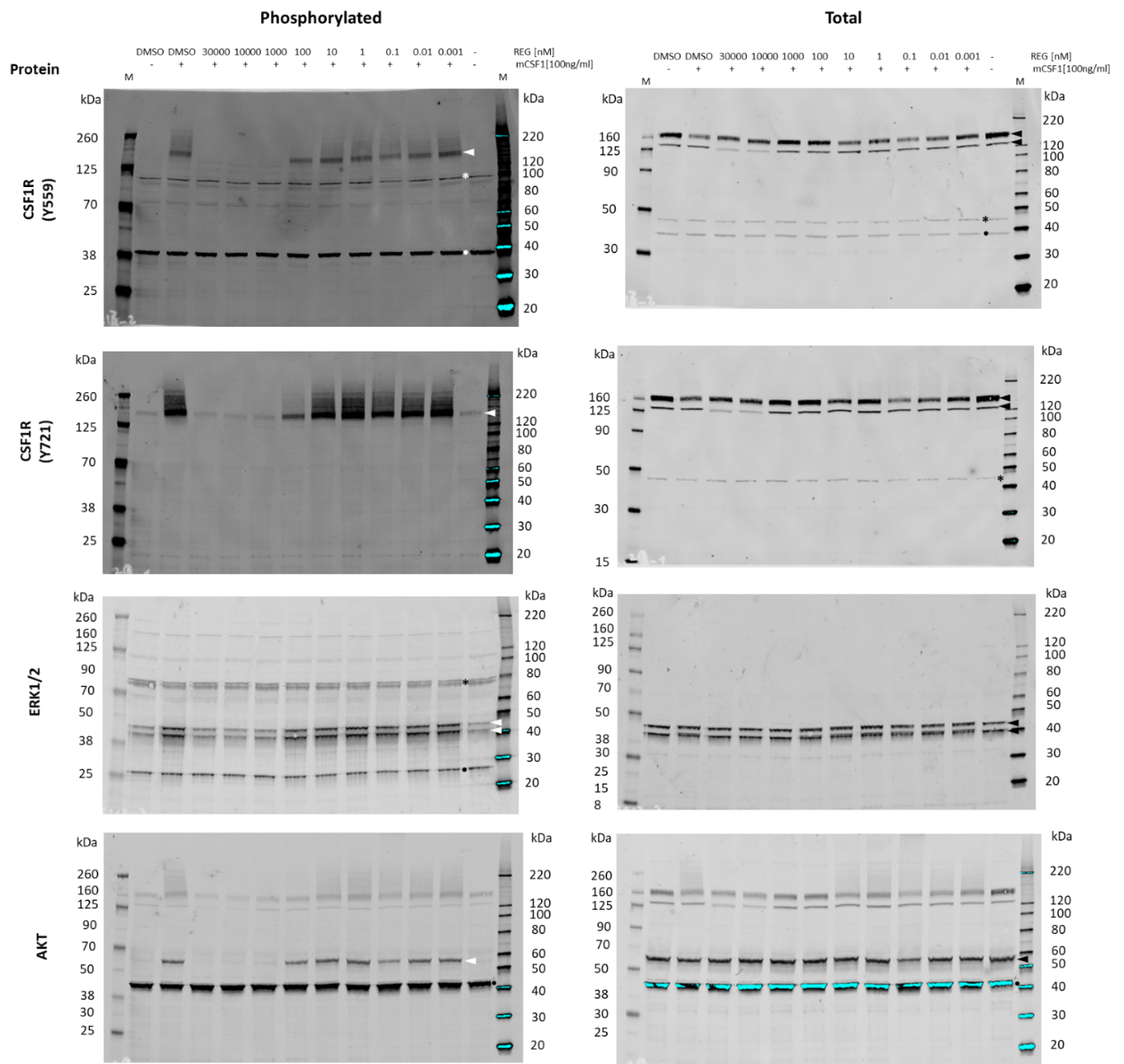

Fig. S3** Entire western blots corresponding to the cropped regions depicted in Fig. 1B. White and black arrow heads indicate diagnostic target proteins. The black/white dots indicate loading control proteins ß-actin and GRB2 (in the ERK1/2 panel). The black asterisks indicate unspecific stains. The bands at the top of the AKT panel are remnants from the CSF1R staining, as the same blot was used for the detection of AKT. Phosphorylated and total proteins were detected on the same blots by using secondary detection antibodies with different fluorescent labels (see Materials and Methods). M: marker

**
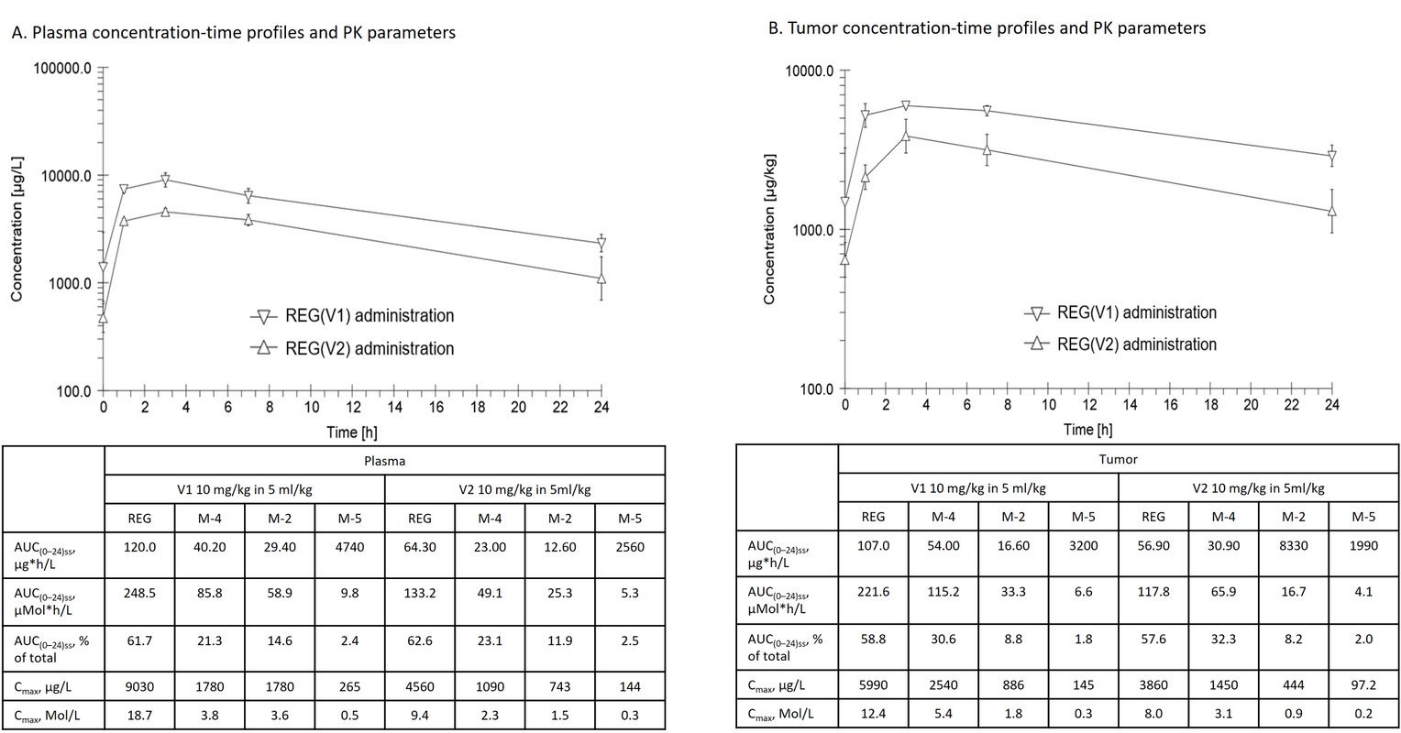
**

**Fig. S4** PK parameters of REG and its metabolites M-2, M-4, and M-5 in **A** plasma and **B** CT26 tumor tissue from BALB/c mice

**
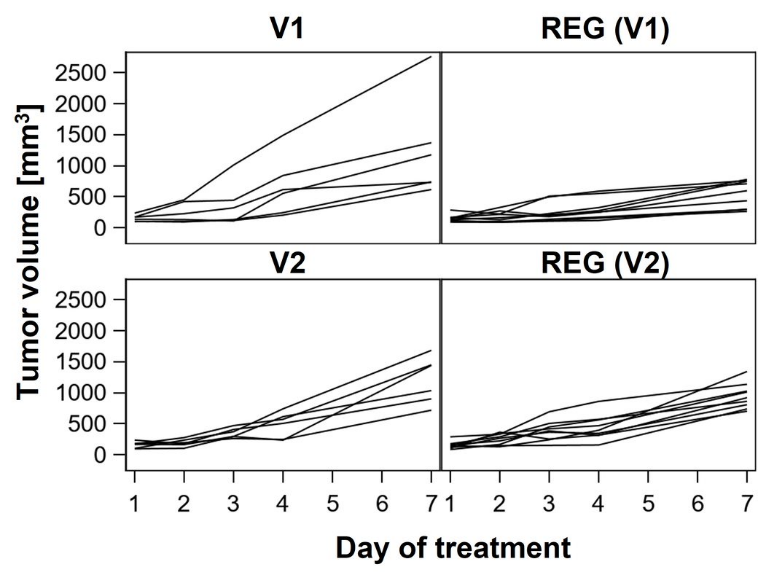
**

**Fig. S5** REG inhibition of CT26 tumor growth, represented by individual tumor growth curves


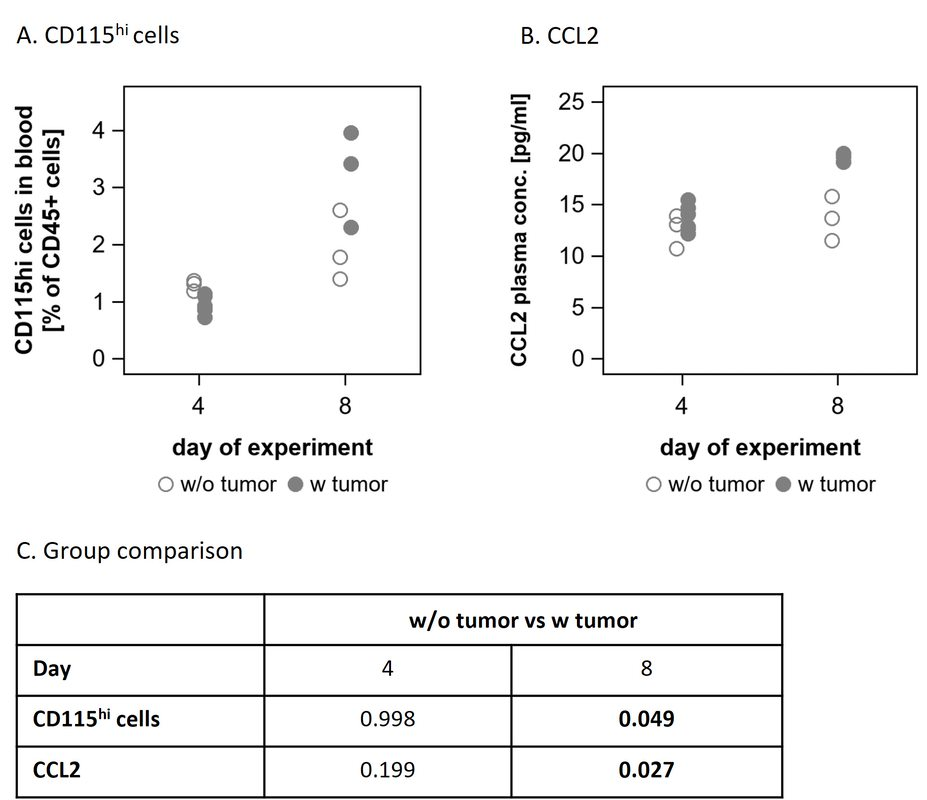
**Fig. S6** CT26 tumors induce elevation of CD115^hi^ cells and CCL2 in PB


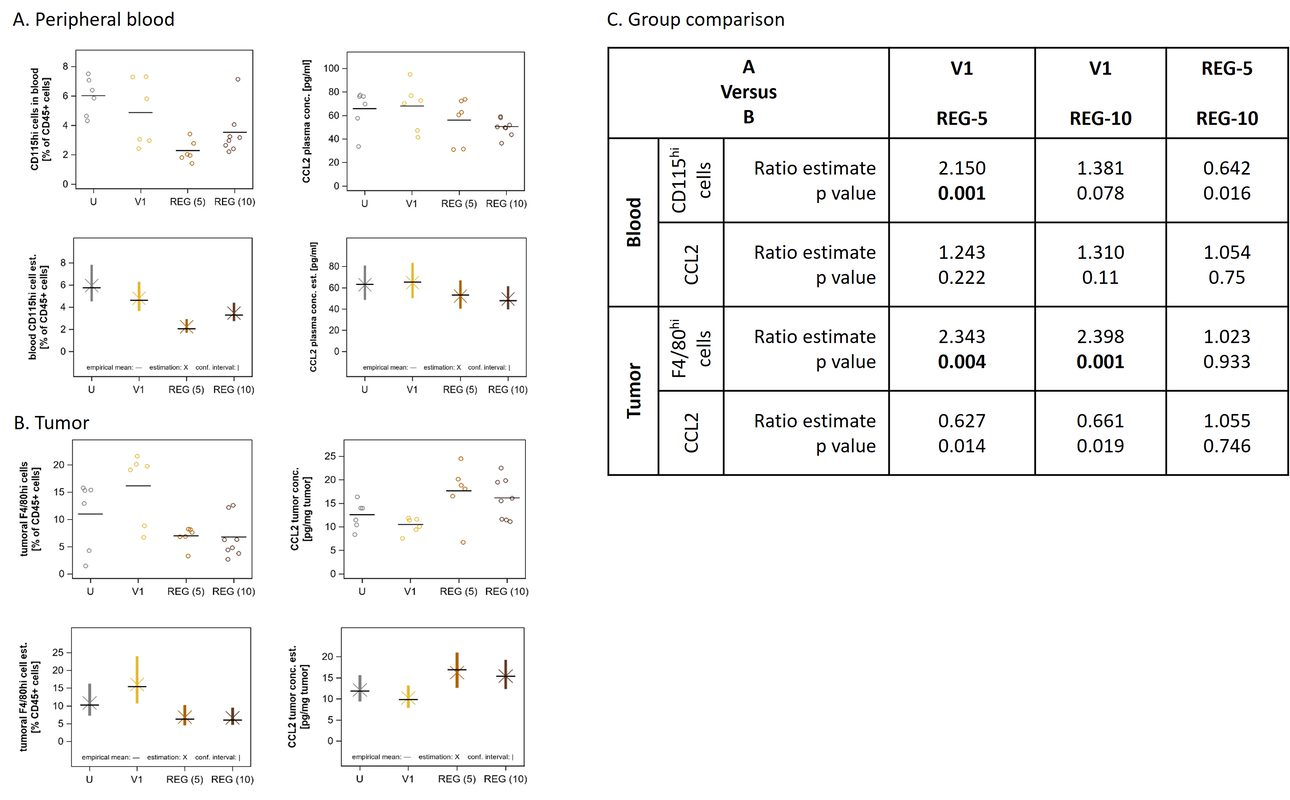


**Fig. S7** Effects of REG on CD115^hi^ cells, F4/80^hi^ cells, and CCL2 in C57BL/6 mice with MC38 CRC tumors at steady state

**
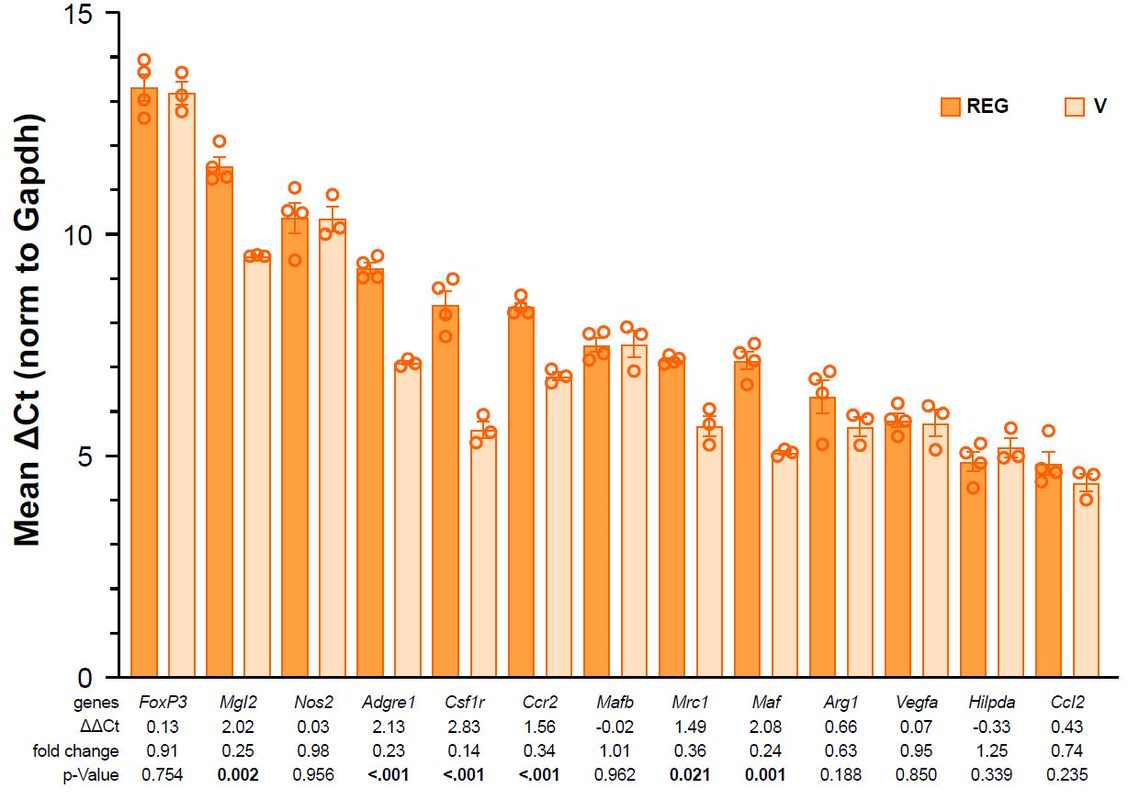
**

**Fig. S8** Effects of REG on macrophage subpopulations by qRT-PCR analysis of selected marker genes
